# Structure Evolution and Properties Modification for Reaction-Bonded Silicon Carbide

**DOI:** 10.3390/ma15248721

**Published:** 2022-12-07

**Authors:** Wei Li, Ge Zhang, Congcong Cui, Jianxun Bao, Conghui Guo, Chuanxiang Xu, Wei Zhang, Wanli Zhu

**Affiliations:** 1 Changchun Institute of Optics, Fine Mechanics and Physics, Chinese Academy of Sciences, Changchun 130033, China; 2 Key Laboratory of Optical System Advanced Manufacturing Technology, Chinese Academy of Sciences, Changchun 130033, China

**Keywords:** reaction-bonded silicon carbide, impregnation, multiphase carbon, microstructure, mechanical performance

## Abstract

Complex structure reaction-bonded silicon carbide (RB-SiC) can be prepared by reactive melt infiltration (RMI) and digital light processing (DLP). However, the strength and modulus of RB-SiC prepared by DLP are not sufficient, due to its low solid content (around 40 vol.%), compared with the traditional fabrication techniques (solid content > 60 vol.%). With this understanding, a new method to improve the properties of RB-SiC was proposed, by the impregnation of composite precursor into the porous preform. The composite precursor was composed of phenolic (PF) resin and furfuryl alcohol (FA). PF and FA were pyrolyzed at 1850 °C to obtain amorphous carbon and graphite into the porous preform, respectively. The effects of multiphase carbon on the microstructure and performance of RB-SiC was studied. When the mass ratio of PF to FA was 1/4, the solid content of RB-SiC increased from 40 vol.% to 68.6 vol.%. The strength, bulk density and modulus were 323.12 MPa, 2.94 g/cm^3^ and 348.83 Gpa, respectively. This method demonstrated that the reaction process between liquid Si and carbon could be controlled by the introduction of multiphase carbon into the porous preforms, which has the potential to regulate the microstructure and properties of RB-SiC prepared by additive manufacturing or other forming methods.

## 1. Introduction

**Figure 1:**
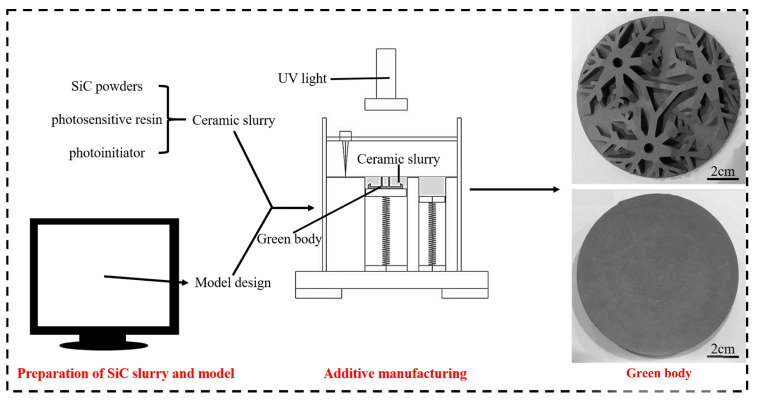
Preparation process of topological structure SiC ceramics.

Reaction-bonded silicon carbide (RB-SiC) with topological or hollow structure have great application prospects in the field of lightweight mirrors and lithography machines. With the development of space remote sensing technology, the aperture of the SiC mirror was required to be larger, which was conducive to improve the resolution of optical system. However, increasing the diameter of the SiC mirror leads to the increase in its mass, which will incur high launch costs and reduce the maneuverability of the probe [1,2,3]. The traditional forming methods are difficult to prepare RB-SiC with highly complex shapes. It is necessary to develop the new methods to meet the needs of the application. At present, DLP is one of the typical additive manufacturing (AM) technologies, which has huge potential to fabricate complex ceramics. The topological structural SiC ceramics was prepared by DLP, as shown in Figure 1. Generally, the low strength (<250 MPa) and low modulus (<300 Gpa) of RB-SiC formed by AM are the common problems, which are the obstacles to application. Therefore, improving the properties of RB-SiC is of great significance [1,2,3].

The typical RB-SiC was obtained after the porous preform infiltrated with liquid silicon (Si). The porous preform was composed of amorphous carbon and α-SiC. During reactive melt infiltration (RMI), liquid Si infiltrated into the porous preform driven by capillary force. The newly formed SiC, named β-SiC, was formed during RMI process. Finally, the pores in the porous preform were filled with the residual Si. Based on the analysis above, the reaction-bonded silicon carbide consisted of α-SiC, β-SiC and Si. Both α-SiC and β-SiC have the properties of high modulus (~450 GPa) and high strength (>350 Mpa) [3,4]. However, the properties of residual Si, such as low modulus (<190 Gpa) and low strength (<100 Mpa), limits the application range of RB-SiC [3,4]. Therefore, it is of great significance to increase the content of SiC in RB-SiC [5,6].

Theoretically, there are two methods to increase the content of SiC, namely, direct method and indirect method [7,8,9,10]. (a) The direct method: increasing the content of SiC in the preform directly by preceramic polymer infiltration and pyrolysis (PIP). In the PIP method, polycarbosilane (PCS) or allylhydridopolycarbosilane (AHPCS) was used as the SiC precursor. PCS or AHPCS transform into SiC at 1000 °C [11,12]. The porous preform was impregnated with PCS solutions and then pyrolyzed at 1000 °C. To increase the content of SiC, PIP process was repeated for several times [11]. Finally, the content of SiC was obviously increased after RMI. However, the direct method is not advantageous in engineering application, due to the disadvantages of a long cycle and high cost. (b) The indirect method: the impregnation method was adopted to introduce the extra amorphous carbon into the porous preform before RMI. Then, the residual Si reacted with amorphous carbon during RMI. The impregnation method is easy to operate, with a low cost and short cycle, and has great potential in engineering application.

Liu [7] prepared the porous preform by additive manufacturing and studied the effect of the asphalt as the precursor of carbon during impregnation. The results showed the strength of RB-SiC could be increased by 20% via impregnation method. Ji-Won Oh [8] enhanced the strength of RB-SiC via the impregnation method, and the phenolic (PF) resin was adopted as the precursor of carbon. The strength of RB-SiC was enhanced from 179 MPa to 270 Mpa. With the increase in the carbon content, the strength increased first, and then decreased due to the phenomenon of pore-clogging. The residual carbon was observed in RB-SiC, which results in limited improvement of strength and modulus for RB-SiC.

Residual carbon was one of the main factors limiting the enhancement of the strength and modulus for RB-SiC. However, there was a lack of research on the elimination of residual carbon in RB-SiC. The essence of pore-clogging was that the reaction rate of Si and carbon was too fast. Fortunately, according to Zou [2], the crystallinity of carbon affects the reaction rate between Si and carbon. Therefore, impregnation and high-temperature pyrolysis were adopted to regulate the content and crystallinity of carbon in the porous preform, to further improve the performance of RB-SiC. During impregnation, furfuryl alcohol (FA) and PF were used as the carbon precursors. The graphitization temperature of the furan ring is lower than that of the aromatic ring [13,14,15]. During high-temperature pyrolysis, the graphite carbon was formed from FA, and the amorphous carbon was formed from PF at 1850 °C. Therefore, the composite carbon sources were introduced into the porous preform by impregnation and high-temperature pyrolysis. The reactivity of carbon is affected by its crystallinity [16,17,18,19]. In this paper, the ratio of graphite carbon to amorphous carbon is controlled to regulate the reaction process between carbon and liquid Si, so as to avoid the phenomenon of pore-clogging. This paper aims at optimizing the strength and modulus of reaction-bonded silicon carbide by regulating composition of the preform. Preforms can be obtained by gel-casting, injection molding or additive manufacturing techniques.

## 2. Materials and Methods

### 2.1. Fabrication of the Specimens

#### 2.1.1. Porous Preform

The porous preform with a solid content of 40 vol.% was prepared, consisting of pyrolytic carbon and α-SiC. The pyrolytic carbon was obtained from monomer (after debinding) and α-SiC as raw materials powder with a diameter of 3 μm. After the polymerization of monomers, the SiC green body was obtained. The high-temperature debinding process was carried out to remove the organic matter in the SiC green body. During debinding, the organic matter was pyrolyzed to amorphous carbon and gas. Gas escaped from the SiC green body and interconnected channels was formed. After cooling to room temperature, the porous preform was obtained, named G0.

#### 2.1.2. RB-SiC

**Figure 2:**
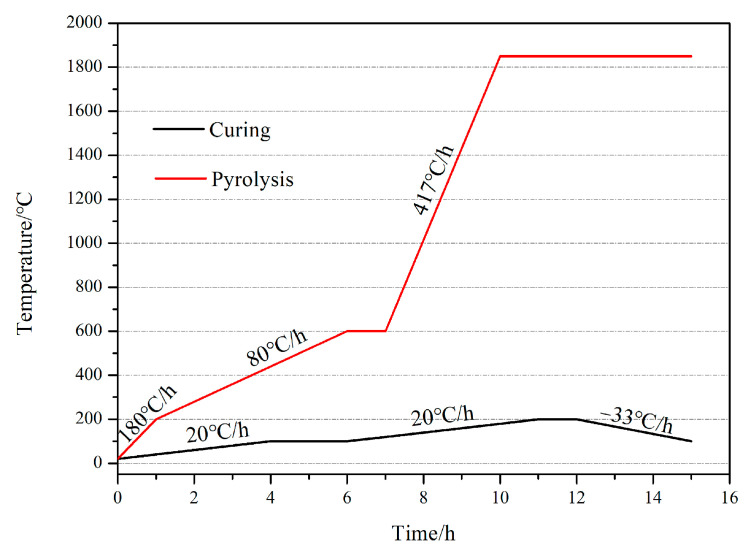
Process program for curing and pyrolysis.

Table 1 shows the nomenclature and compositions of all the specimens in this experiment. The carbon precursor solution was composed of oxalic acid as curing agent (Tianjin Zhiyuan Chemical Reagent Co., Ltd., Tianjin, China), FA (Shanghai Zhanyun Chemical Co., Ltd, Shanghai, China) and PF (Changchun Shengda Insulation Material Co., Ltd., Changchun, China). The preforms were immersed in the carbon precursor solution for 10 h before drying and curing. The carbon precursor solutions were divided into three groups, according to the PF content of 10 wt.%, 20 wt.% and 30 wt.%. After curing, high-temperature pyrolysis was carried out at 1850 °C in vacuum. The curing and pyrolysis process curves are shown in Figure 2. After cooling to room temperature, the specimens of G10, G20 and G30 were obtained according to the content of PF.

The Si powders were placed on the surface of the preforms, and when the temperature was raised to the melting point of Si, the liquid Si penetrated into the preforms. The RMI program was shown in our previous work [6]. Finally, the sintered specimens were obtained and named P10F90, P20F80 and P30F70, according to the content of PF in carbon precursor solution. In RMI, P0F0, as the standard group without impregnation and high-temperature pyrolysis, was treated under the same conditions as P10F90, P20F80 and P30F70.

### 2.2. Characterization of the Specimens

The microstructures of the preforms and RB-SiC were observed by optical microscopy (Olympus Corporation, Tokyo, Japan) and second electron microscopy (Phenom-World, Eindhoven, The Netherlands). The structure of carbon after high-temperature pyrolysis was analyzed by Raman spectrometer.

An X-ray diffractometer (XRD, Bruker/D8FOCUS, Bruker Instrument Co., Ltd., Karlsruhe, Germany), which was equipped with a Cu Kα radiation source, was used to obtain the phase composition of specimens. The Raman spectrometer (HOOKE P300, HOOKE Instruments Ltd., Changchun, China) was used for Raman measurements. The bulk density and apparent porosity were measured according to Archimedes principle. The flexural strength was measured by three-point bending method with a loading rate of 0.5 mm/min. The elastic modulus was calculated by stress–strain curve. The residual Si was counted by image J (version 1.8.0) software.

## 3. Results and Discussion

In this paper, the composite carbon sources were introduced into the capillary channel by impregnation. Based on the reactivity of carbon is affected by the structure, the reaction rate between liquid Si and carbon is regulated by changing the ratio of graphite carbon to amorphous carbon. Finally, the RB-SiC with the best properties was obtained, with the lowest content of residual carbon and silicon.

### 3.1. Fabrication of Composite Carbon in Capillary Channel

**Figure 3:**
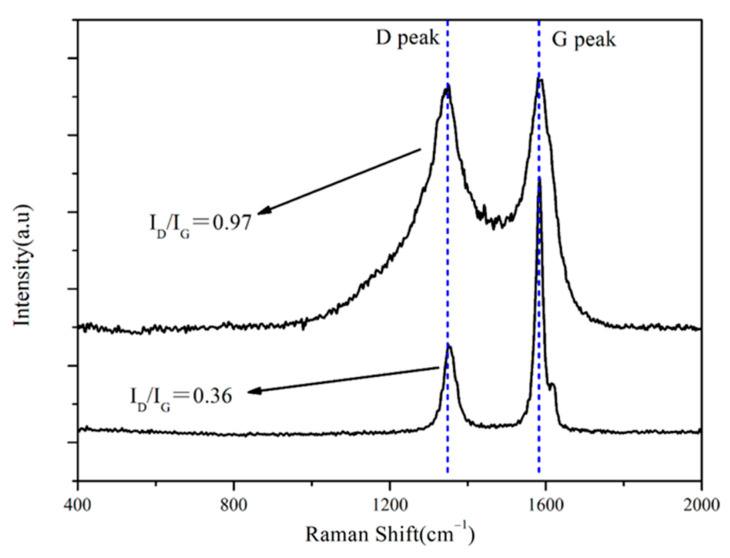
Raman analysis for G10.

The key of this study is to prepare multiphase carbon in the capillary channel. Raman spectroscopy is one of the effective methods to detect the structure of carbon [20,21]. D peak and G peak are the typical characteristic peaks of carbon, located at wave numbers 1350 cm^−1^ and 1580 cm^−1^, respectively. In general, the ratio of the intensity corresponding to the D peak to G peak, namely I_D_/I_G_, represents the crystallinity of the carbon. The graphite carbon was obtained, when the value of I_D_/I_G_ was less than 0.4 [20]. Figure 3 shows the Raman spectrum analysis of G10. It can be seen that graphite carbon and amorphous carbon were introduced into the capillary channel of the porous preform after impregnation and high-temperature pyrolysis. The D peak represents the lattice defect, and the intensity increases with the increase in defects existed in the carbon. At 1850 °C, the value of was I_D_/I_G_ 0.97, indicating that defects existed in the carbon, which is similar to amorphous carbon. The value of I_D_/I_G_ was 0.36, indicating the graphitization process had occurred at 1850 °C, as shown in Figure 3. In the pyrolysis of the furan ring, the carbon–oxygen double bond was broken, and the molecular chain was reorganized to form a new six-numbered carbon ring. According to XIA’s report [13], the newly formed six-numbered ring was more active than aromatic compounds, due to it being easier for the hydrogen atoms to escape or be replaced. Therefore, the graphite carbon and amorphous carbon were introduced into the capillary channel of the porous preform from FA and PF at 1850 °C, respectively.

**Figure 4:**
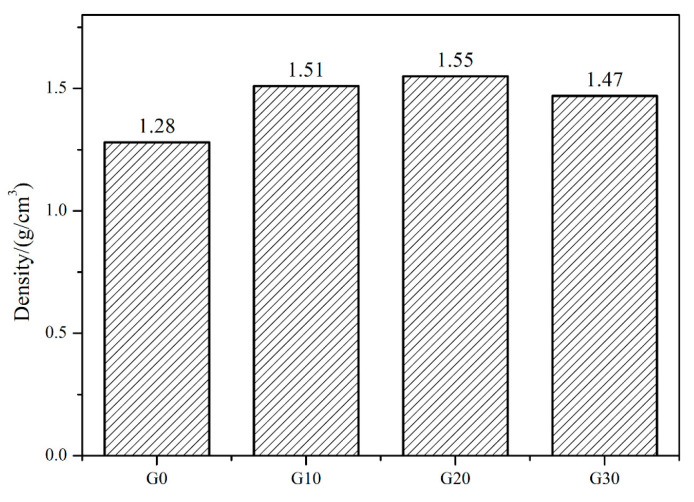
The density of G0, G10, G20 and G30.

The structure of carbon pyrolyzed from PF is different from that of FA. Graphite carbon and amorphous carbon were introduced into the capillary channel of the porous preform. The reaction rate between amorphous carbon and liquid Si was fast, and a large amount of SiC was formed in a short time. The capillary channel was closed by new-formed SiC, which prevented the subsequent liquid Si infiltrating the porous preform. Finally, the residual carbon and pores were observed in RB-SiC. However, when the amorphous carbon was replaced by graphitic carbon, the capillary channel was not closed during RMI, due to the low reaction rate between graphitic carbon and liquid Si. The porous preform was filled with liquid Si. However, it is possible that graphitic carbon remains unreacted after the RMI program. Finally, the graphitic carbon was detected in RB-SiC as the impurities. In order to minimize the residual carbon and residual Si in RB-SiC, RMI was carried out with different ratios of carbon sources. From G10 to G30, the concentration of PF increased from 10 wt.% to 30 wt.%. Figure 4 shows the density of G0, G10, G20 and G30. With the increase in the concentration of PF, the density of the porous preform increased gradually. By comparing G30 with G20, it can be found that when the concentration of PF is 30 wt.%, the density of the porous preform decreased. PF was dissolved in FA to form the composite precursor solution. The viscosity of the composite precursor solution increased, with the increase in PF concentration. The good fluidity of the precursor solution is beneficial to the impregnation process of the porous preforms. In G0, G10, G20 and G30, the density is proportional to carbon content in the porous preform. However, the composite precursor solution obtained the worse fluidity and the content of carbon decreased, when the concentration of PF increased to 30 wt.%. When the concentration of PF was 20 wt.%, the carbon content in the porous preform reached the maximum, which was beneficial to enhance the content of reinforced phase SiC after RMI.

**Figure 5:**
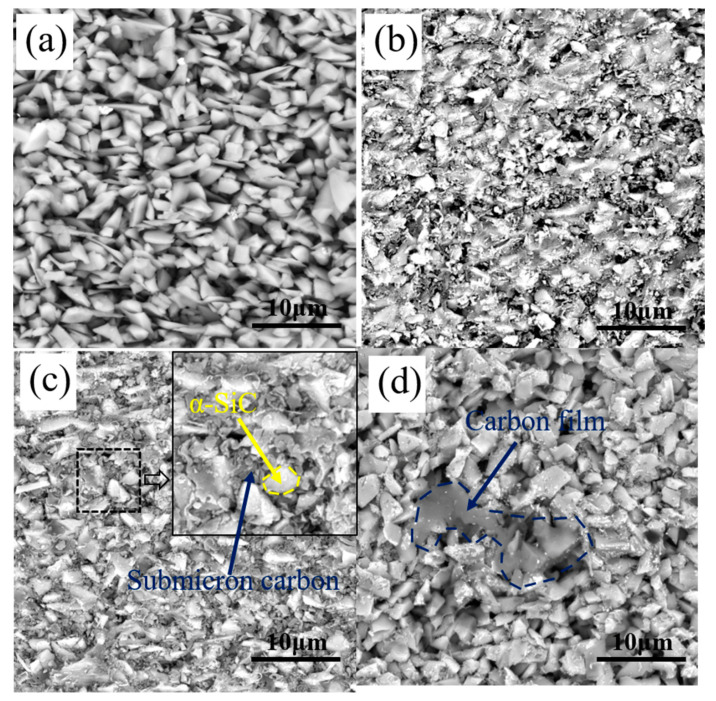
The microstructure of: (**a**) G0, (**b**) G10, (**c**) G20 and (**d**) G30.

Figure 5 shows the microstructure of G0, G10, G20 and G30. The porous preform was composed of pores, carbon and α-SiC after debinding. Carbon was obtained by the pyrolysis of organic matter, such as monomer and cross-linker at a high temperature. Due to the low carbon yield of organic matter, the content of carbon in G0 was low. As shown in Figure 5a, the α-SiC particles are overlapping each other and a large number of micro-scale pores existed in G0. After the RMI process, the liquid Si nucleated and grew at the pores, and “Si islands” were formed, which was detrimental to the properties of the RB-SiC. By comparing the microstructure of G10 and G20, it can be found that a large amount of pyrolysis carbon was formed around the α-SiC. As shown in Figure 5c, submicron carbon particles filled the pores, reducing the diameter of the pores. In addition, the comparison between G10 and G20 shows that the size of the pores decreased with the increase in the content of PF. However, it is found that two kinds of carbon existed in G30: one is the submicron carbon particles, and the other is the carbon films, as shown in Figure 5d. With the increase in the concentration of PF, the viscosity of the precursor solution increased, which was not conducive to the impregnation process of the porous preforms. Therefore, when the concentration of PF increased to 30 wt.%, the carbon content of the porous preform decreased, as shown in Figure 4 and Figure 5d. The morphology of carbon in G10, G20 and G30 was related to the content of FA. After high temperature pyrolysis at 1850 °C, PF was pyrolyzed to the amorphous carbon, named, PF-amorphous carbon. The FA was pyrolyzed to amorphous carbon at 1000 °C. The atoms of amorphous carbon were rearranged to form graphite carbon at 1850 °C. The density of graphite carbon is higher than that of amorphous carbon, so physical shrinkage would occur during the graphitization of amorphous carbon. The shrinkage process brought tensile stress to the PF-amorphous carbon. Figure 5b,c shows the tensile stress that caused the PF-amorphous carbon to be broken into small size carbon particles. The tensile stress decreased with the decrease in FA content. The tensile stress in G30 was not sufficient to break the PF-amorphous carbon. Therefore, the carbon films were observed, as shown in Figure 5d. Therefore, the multiphase carbon changed the structure of capillary channel in the porous preform. The newly formed capillary channel was more conducive to the infiltration of liquid Si.

**Figure 6:**
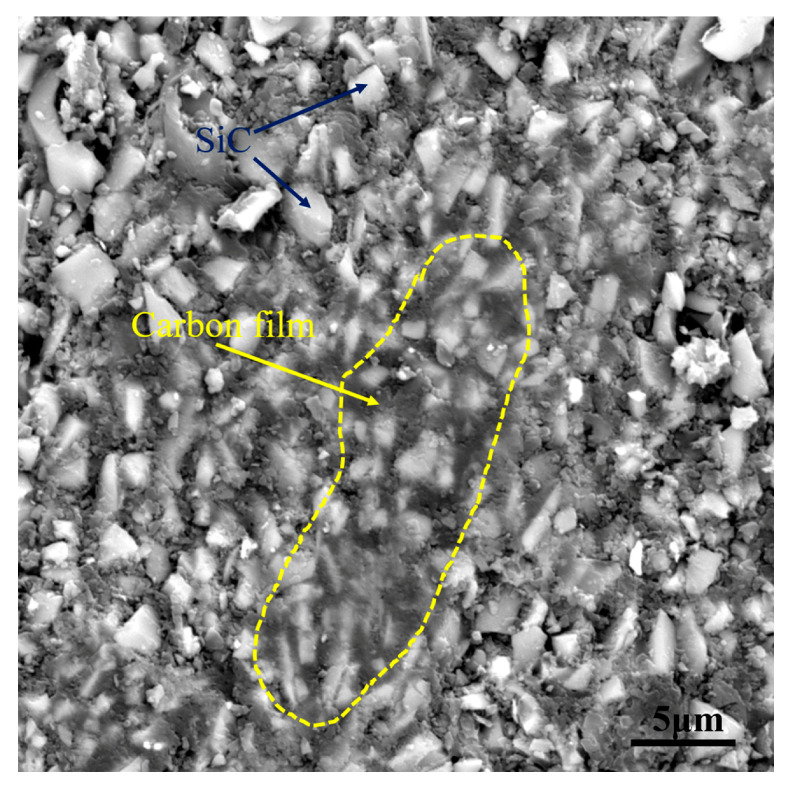
The microstructure of PF-100.

From the analysis above, it can be seen that the multiphase carbon, which was obtained by the pyrolysis of the PF and FA, has the effect of changing the structure of the capillary channel. To verify the above inference, comparative experiment was supplemented. The porous preform was impregnated with PF solution only and pyrolyzed at 1850 °C to serve as the verification group. The verification group was named PF-100. Figure 6 demonstrates the microstructure of PF-100. Both amorphous carbon and graphite carbon existed in G20. Unlike G20, the carbon in PF-100 was amorphous carbon. As shown in Figure 6, the amorphous carbon in PF-100 existed in the form of carbon film. The capillary channels were filled with carbon film, which was not conducive to the infiltration process of liquid Si. Therefore, the comparison between Figure 5c and Figure 6 shows that the composite precursor is conducive to changing the structure of capillary channels.

### 3.2. Mechanism of Multiphase Carbon during RMI

**Figure 7:**
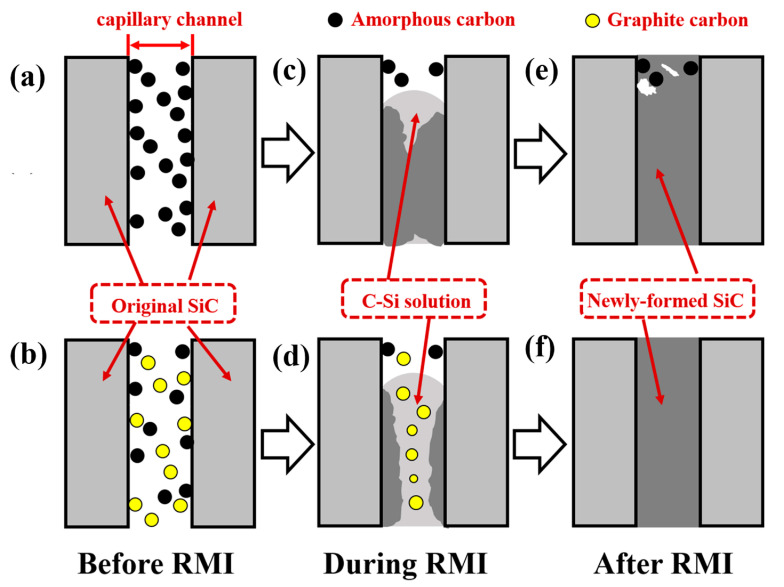
Schematic of reaction process between carbon and liquid Si: (**a**) the amorphous carbon in capillary, (**c**) the reaction between amorphous carbon and liquid Si, (**e**) the phenomenon of pore-clogging; (**b**) the multiphase carbon in capillary, (**d**) the reaction between multiphase carbon and liquid Si, (**f**) the phenomenon of the pore-clogging was avoided.

As reported in Refs. [7,8,9,22,23], the impregnation method could improve the performance of the final body via regulating the composition of the porous preform. However, when more carbon was introduced into the porous preform, the unreacted carbon was detected in RB-SiC due to the phenomenon of pore-clogging. The residual carbon as the impurities weakened the properties of sintered body. According to Song’s result [24], the reason for pore-clogging was that the reaction rate between liquid Si and amorphous carbon was too fast. The reaction process of carbon and liquid silicon follows the mechanism of dissolution and precipitation. Liquid silicon penetrates into the porous preform along the capillary channel due to capillary forces. The carbon was dissolved in liquid silicon, forming the carbon–silicon (C-Si) solution. When the content of carbon in liquid silicon was supersaturated, the reaction product silicon carbide precipitated in the inner wall of the capillary channel. With the progress of the reaction, the diameter of the capillary channel decreased gradually. When the capillary channel was completely filled with newly formed silicon carbide, the infiltration process of liquid silicon into the porous preform was terminated, as shown in Figure 7a,c,e. This was the main reason for the presence of residue carbon and even pores in RB-SiC after RMI. As in Zhang’s report [17], the reactivity of carbon was influenced by structure, crystallinity and size. Therefore, the reaction rate between liquid Si and carbon could be regulated by the crystallinity of carbon, so as to eliminate the residual carbon. In this paper, the multiphase carbon was used to avoid the phenomenon of pore-clogging, to improve the properties of RB-SiC. The mechanism of multiphase carbon is shown in Figure 7b,d,f. Compared with amorphous carbon, graphite carbon is less reactive [2]. Therefore, the precipitation rate of newly formed silicon carbide was reduced by graphitization. During the RMI process, this method was conducive to the infiltration of liquid silicon. Finally, the dense Si/SiC materials were obtained without residual carbon or pores, as shown in Figure 7b.

### 3.3. Composition and Performance for RB-SiC

**Figure 8:**
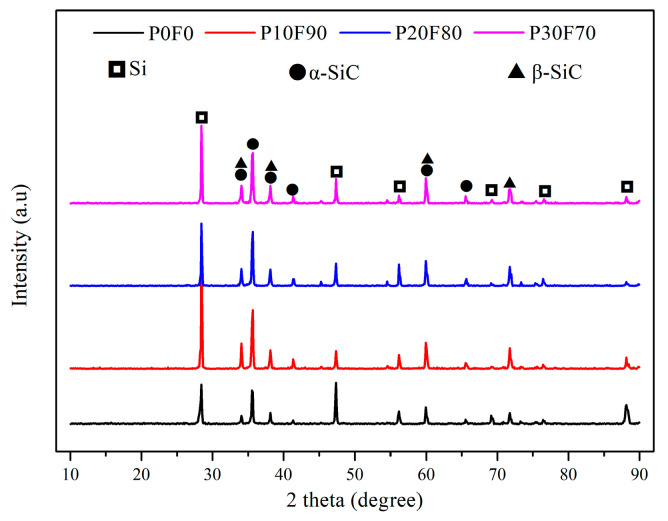
The XRD patterns of P0F0, P10F90, P20F80 and P30F70.

After RMI, the RB-SiC of four groups were named as P0F0, P10F90, P20F80 and P30F70, corresponding to G0, G10, G20 and G30, respectively. The XRD patterns of P0F0, P10F90, P20F80 and P30F70 are demonstrated in Figure 8. The final body consisted of Si, α-SiC and β-SiC. The properties of RB-SiC are affected by the content of residual Si after RMI. It can be found that the Si content of P10F90 was lower than P0F0, according to the analysis of diffraction peaks of Si from crystal plane (111), (220) and (311) corresponding to the 2θ located at 28.44°, 47.30° and 56.12°, respectively. Compared with P0F0, the relative intensity of the diffraction peaks of Si from the crystal plane (220) and (311) in P10F90 decreased significantly, indicating that the Si from the crystal plane (220) and (311) had higher reactivity with carbon. However, the diffraction peak of Si from crystal plane (111) for P10F90 are more intense than for P0F0, which may be caused by the measurement error due to the uneven microstructure of the specimens. The impregnation of composite precursor is one of the effective ways to reduce the content of residual Si. In addition, the typical characteristic peak of carbon was not detected from the XRD patterns of P10F90, P20F80 and P30F70, which indicated that the multiphase carbon could avoid the phenomenon of pore-clogging.

**Figure 9:**
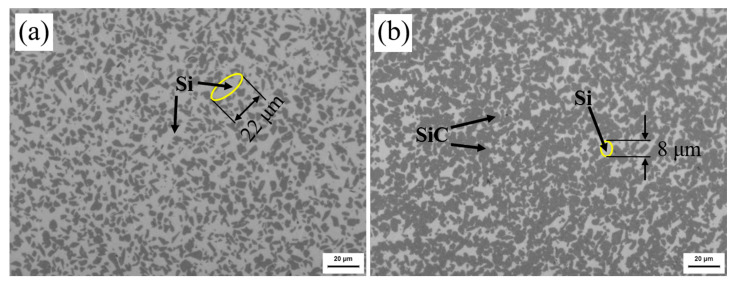
The microstructure of RB-SiC for: (**a**) P0F0, (**b**) P20F80.

The metallographic microstructure of P0F0 and P20F80 are shown in Figure 9. In Figure 9, the bright and the dark represent Si and SiC, respectively. When the concentration of PF in the composite precursor solution was 20 wt.%, the density of the porous preform reached maximum, as shown in Figure 4. Compared with P0F0 and P20F80, the increase in SiC content and residual carbon due to multiphase carbon was not observed in RB-SiC. Without multiphase carbon, the size of residual Si was 22 μm, as shown in Figure 9a. Liquid Si reacted with multiphase carbon in P20F80, accompanied by volume expansion. The remaining Si filled the capillary channel, and eventually, dense RB-SiC was obtained. Therefore, the size of residual Si was related to the capillary channel. The introduction of multiphase carbon reduced the size of residual Si to 8 μm, as shown in Figure 9b.

**Figure 10:**
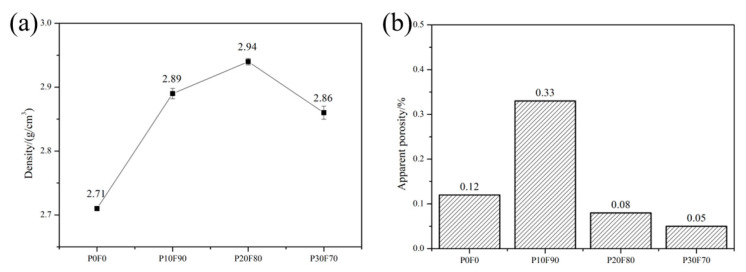
Compare and demonstrate the performance of P0F0, P10F90, P20F80 and P30F70: (**a**) bulk density, (**b**) apparent porosity.

The bulk density of SiC is obviously higher than Si. Therefore, the bulk density of RB-SiC would definitely be increased with the increase in SiC content. Comparing P10F90 with P0F0, the impregnation method increased the density of RB-SiC by 6.6%. When the concentration of PF was 20 wt.%, the density of P20F80 reached the maximum with a value of 2.94 g/cm^3^. However, the bulk density of P30F70 was only 2.86 g/cm^3^, as shown in Figure 10a. Due to the limited solubility of PF in FA, when the concentration of PF was 30 wt.%, the viscosity of the composite precursor solution was increased, and the efficiency of impregnation was reduced. The density of RB-SiC was consistent with that of the porous preform, as shown in Figure 4 and Figure 10a. The apparent porosity is one of the key factors affecting the mechanical properties of ceramics. In the development of precision ceramics, the apparent porosity should be reduced as much as possible. The pores on the surface of RB-SiC lead to stress concentration. As the stress increases, cracks will be formed and multiplied. Unlike plastics, ceramics are remarkably brittle. Once the cracks appear, the defect will grow and multiply until the rupture occurs. Figure 10b shows that the apparent porosity values of P0F0, P10F90, P20F80 and P30F70 are lower than 0.4%. When the concentration of PF was 10 wt.%, RB-SiC obtained the maximum apparent porosity of 0.33%, which might be caused by the errors of grinding and polishing.

**Figure 11:**
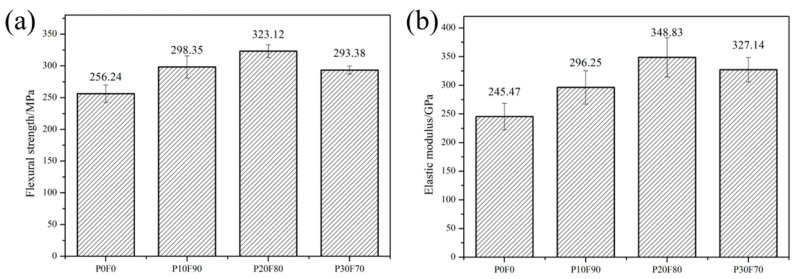
Comparing and demonstrating the performance of P0F0, P10F90, P20F80 and P30F70: (**a**) flexural strength, (**b**) elastic modulus.

The aim of this paper is to study the effect of multiphase carbon on strength and modulus of RB-SiC. Figure 11 demonstrates the results of strength for P0F0, P10F90, P20F80 and P30F70. The increase in the concentration of PF in the composite precursor solution indicated that the amorphous carbon content in the capillary channel was increased. According to Refs. [17,18,19], the reactivity of amorphous carbon is higher than that of graphite carbon. The reaction rate of Si with carbon could be regulated by the ratio of graphite carbon to amorphous carbon. Compared P10F90 with P0F0, it could be found that the flexural strength of RB-SiC was enhanced to 16.43% via impregnation method. As shown in Figure 11a, when the concentration of PF was 20 wt.%, the RB-SiC obtained the maximum flexural strength and the value of the strength was 323.12 MPa. However, the flexural strength of P30F70 was 293.38 MPa, which was lower than P20F80. The factors affecting the strength of ceramics include the apparent porosity, the content of reinforcing phase and impurities. XRD analysis shows that the composition of P30F70 is consistent with that of P20F80 and the impurities are not detected in P30F70. P30F70 has the lowest apparent porosity in Figure 10a. Therefore, the main factor affecting the strength of RB-SiC is the content of SiC. The content of SiC can be roughly calculated according to the density of RB-SiC. RB-SiC consisted of Si and SiC. The density of Si is 2.35 g/cm^3^ and the density of SiC is 3.21 g/cm^3^. The results show that volume fraction of SiC in P20F80 and P30F70 is 68.60 vol.% and 59.30 vol.%, respectively. In addition, the content of SiC is one of the main factors affecting the elastic modulus of RB-SiC. The elastic modulus of Si and SiC is 190 GPa and 470 GPa [4], respectively. Among P0F0, P10F90, P20F80 and P30F70, the elastic modulus of P20F80 was the largest, which was 348.83 GPa. The elastic modulus could be increased to 42.11% by the multiphase carbon in the porous preform. The elastic of P30F70 was higher than that of P10F90, as shown in Figure 11b. This might be due to the surface defects of P10F90 that reduced the integrity of RB-SiC, as shown in Figure 10b.

## 4. Conclusions

The multiphase carbon has the function of regulating the reaction rate between liquid Si and carbon. The multiphase carbon was introduced into the porous preform via the high-temperature pyrolysis of composite precursor. The composite precursor solution was composed of PF and FA. The microstructure and mechanical properties were regulated. The specific conclusions are as follows.

1. Porous preform

The composite precursor changed the structure of capillary channel, making the new-formed capillary channel more conducive to the infiltration process during RMI. When the concentration of PF was 20 wt.%, the submicron carbon particles refined the capillary channel, which was beneficial to reduce the dimension of Si island in reaction-bonded silicon carbide.

2. The microstructure of RB-SiC

The impregnation of composite precursor enhanced the content of SiC. Multiphase carbon regulated the reaction rate between liquid Si and carbon, and it avoided the phenomenon of pore-clogging. The residual carbon was not observed in RB-SiC.

3. The performance of RB-SiC

Composite precursor impregnation could improve the strength and modulus of RB-SiC. RB-SiC obtained the best performance, when the concentration of PF was 20 wt.%. The bulk density, strength and modulus of RB-SiC were increased by 8.49%, 26.10% and 42.11%, respectively.

## Figures and Tables

**Table 1:** Nomenclature used to distinguish the prepared preforms and RB-SiC.

Group	Composition of Carbon Precursor		Impregnation and High-Temperature Pyrolysis	Named	
	FA/wt.%	PF/wt.%		Before RMI	After RMI
1	90	10	✓	G10	P10F90
2	80	20	✓	G20	P20F80
3	70	30	✓	G30	P30F70
4	0	0	✗	G0	P0F0

## Data Availability

The data presented in this study are available on request from the corresponding author.
